# Hypnotism in Hysterical Paralysis

**Published:** 1856-04

**Authors:** 


*Hypnotism in Hysterical Paralysis.* By JAMES BRAID, Esq., of Manchester. ("*Association Medical Journal*," *Sept.* 14, 1855.)

We would wish to direct especial attention to the facts and arguments contained in this article, for, as we take it, the arguments are as sound as the facts remarkable. What *hypnotism* is, or wherein it differs from mesmerism, we shall leave Mr. Braid to tell us in his own words, for we can add nothing to the clearness of his description.

"It is no doubt known to many," he says, "that in 1841, I entered upon the experimental investigation of the phenomena of mesmerism. I did so as a decided sceptic in every respect, resolved, if possible, to discover and expose the trick by which certain phenomena then being exhibited in Manchester, were accomplished. I very soon discovered, however, that there was a certain amount of truth, mixed up with what I believed to be error; and I therefore resolved to attempt to separate the former from the latter.

"I was soon enabled to demonstrate that, by certain processes some individuals were able, by their own unaided efforts, to throw themselves into an analogous state with those who were subjected to the mesmerising processes. The most speedy and certain mode for accomplishing this was causing the subject to maintain a steady fixed gaze at any inanimate object, placed a little above the forehead, but so as to be seen distinctly by both eyes; the subject at the same time concentrating his undivided attention upon said unexciting act. This was at once a most important step, as it proved the influence to be *subjective*, or a personal influence existing within the patient's own body, and not depending upon any influence *ab extra*, proceeding from the body of another human being. This inference was still farther supported by the fact that the varied nature and quality of the object gazed at seemed in no way materially to alter the subsequent results; whilst, in highly impressible subjects, it was proved that the results depended so much upon the expectant idea in the mind of the subject, that any physical combination of circumstances whatever to which they were told to direct their attention, with the assurance that they would be thereby sent to sleep, was followed by sleep; whilst the next moment they might comply with the same directions without going to sleep at all, if persuaded by the suggestion of a second party, or by a pre-existing idea in his mind to the effect that now the agency was to be inoperative, from some supposed change in the existing circumstances. It was moreover ascertained that, with some very susceptible subjects, the mere idea and belief that some particular process was going on at a distance, to send them to sleep, was sufficient to produce such result, even when no such process was taking place: and farther, in reference to the alleged power of the will of the operator to affect subjects, either near at hand or at a distance, after having carefully investigated the subject, I am warranted to state, as the result of my experience, that I have never found any influence whatever to be exerted over the patient by my *silent willing;* but they seemed very quick to catch suggestions from the manners, looks, tones of voice, or physical manipulations of the operator; and to become affected according to the purport of what they *inferred* to be the *will* and *intention* of the operator, and that even when he might be *willing* the *very reverse.* In short, my experience went to prove that the real efficient agency of all the different processes was merely as aids to assist the patient to induce in himself a state of mental abstraction or fixity of attention, in which the powers of his mind should be so absorbed by a fixed idea or given train of thought as, for the nonce, to render him dead or indifferent to all other considerations and influences which did not harmonize with the dominant idea in his (the patient's) mind at the time.

"As a very strong corroboration of the correctness of this view of the subject, I may state the fact that, from the difficulty of fixing the attention of idiots, all my attempts to hypnotise them have been unsuccessful, notwithstanding I have made many persevering attempts to do so.

"Again, in my experimental inquiry into *The Power of the Mind over the Body*, which was published in 846, as the result of a laborious set of experiments, I was enabled to demonstrate that a sustained act of attention, directed to any part of the body, was followed in a few minutes by a change or modification in the physical function of the organ or part so regarded; the general result being an exaltation of function; but, curiously enough, with many individuals the very reverse result might ensue, from a dominant idea to that effect having existed in the mind of the patient previously, or from its being strongly imprinted on his mind at the moment by an audible suggestion from another person, in whose prediction he could repose confidence. The more vivid the imagination and fixed the attention of the subject to the expected result, the more certainly and vividly were the phenomena realized; and after the processes for inducing what I call the hypnotic state, it was found that these physical phenomena could be produced, through the mental influences of the subjects, with far more certainty, celerity, and intensity, than in the ordinary waking condition. I therefore adopted the term hypnotism, or nervous sleep, to designate this peculiar condition of the nervous system, into which it could be thrown by artificial contrivance, and which differed in several respects from common sleep, as well as from the ordinary waking condition. In fact, hypnotism comprises, not one state, but rather a series of stages or conditions, varying in every conceivable degree, from the slightest reverie, with high exaltation of the function called into action, on the one hand, to intense nervous coma, of entire abolition of consciousness and voluntary power, on the other; whilst, from the latter condition, by very simple but appropriate means, the patient is capable of being speedily partially restored, or entirely roused to the waking condition. By this means, I maintain that the operator does not communicate any surcharge of a magnetic, odylic, electric, or vital fluid or force, from his own body to that of the patient, as the real and efficient cause of the phenomena which follow, in altering or modifying physical action, or curing disease; but I hold that he acts merely as the engineer, by various modes, exciting, controlling, and directing the *vital forces within the patient's own body*, according to the laws which regulate the reciprocal action of mind and matter upon each other in the present state of our existence."

Mr. Braid enters more fully into this part of his subject, and suggests several new terms, but we have said enough to make the cases intelligible which we are about to relate, and this is our sole purpose at present. These cases comprise four of hysterical spasm or paralysis (of which we take two), and one of impaired vision, also no doubt of an hysterical character. These cases we leave to tell their own story, merely premising that Mr. Braid does not always depend upon hypnotism, to the exclusion of the ordinary modes of treatment.

Case 17.—In the spring of 1842, a girl was brought to me, under the following circumstances. She had been suddenly seized with violent tonic spasms of the hand and arm, so that her hand was rigidly clinched, the wrist flexed on the arm, and the arm flexed on the humerus, attended with considerable pain. Most of the servants, male as well as female, in a large hotel, had exerted their utmost strength without being able to open the hand or extend the arm. A highly respectable surgeon was now sent for, who prescribed medicine internally, and the application of a large blister on the nape of the neck. There being an aggravation of symptoms, I was consulted, when I immediately recognized this as an hysteric case, for which I hypnotized her, and then, by gently titillating the skin along the course of the extensor muscles, they were immediately called into action, and the morbid action of the flexors at the same time withdrawn, by which simple means, by *art*, and without the slightest effort, I was enabled, in a few minutes, to effect what had resisted the strongest efforts of powerful men to accomplish; for the hand was opened, and the wrist and arm extended, and the patient cured in a few minutes; and she never had any subsequent attack of the sort.

Case 19. — On the 11th of August, 1853, a young lady, 23 years of age, was sent to me from Berwick-upon-Tweed, by Dr. Johnston of that city. The following is the history of this very interesting case. Four years previously she had been seized with a paralytic dragging of the left leg, which became worse and worse, notwithstanding the most assiduous efforts of Dr. Johnston, who is a most experienced and scientific physician, aided by the opinion and advice of Sir B. Brodie, who had been corresponded with on the case by Dr. Johnston. Four months having elapsed without improvement, she was taken to Edinburgh, for a consultation with Professor Syme, who examined her spine with great care, said he found no disease there, and hoped she would recover ultimately, also several years might elapse before such event took place; and that he could only in the meantime, recommend attention to her general health, with exercise in the open air, by riding on a donkey. This was persevered in for ten months more, without the slightest improvement, when the patient was taken to London, to have the benefit of the personal examination and advice of Sir B. Brodie. Still no improvement ensued for months, and at length all treatment was abandoned. After this patient had been in this condition for twenty months, and after all treatment had been abandoned as quite inefficacious in her case, she at length gradually recovered the use of her limbs. From that period she had occasional threatenings of a return of her old complaint; and, in the summer of 1852, she frequently felt as if her legs were being galvanized. In February, 1853, she had a return of her paralytic affection, which continued for a fortnight; and, at the end of April 1853, she had another seizure, which had obstinately resisted all the best directed efforts of her old and tried friend, Dr. Johnston, for four months, when he sent her to me, to try the effect of hypnotism; which he had been induced to do from having read some remarks on the subject contained in my paper on "Hypnotic Therapeutics," published in the July number of the *Monthly Journal of Medical Science*, for 1853. The following is the passage which impressed Dr. Johnston with the belief that hypnotism would be the remedy for his patient; and he immediately recommended it to the parents, and wrote to me accordingly, and sent his patient hither.

"The most striking cases of all, however, for illustrating the value of the hypnotic mode of treatment, are cases of hysteric paralysis, in which, without organic lesion, the patient may have remained for a considerable length of time perfectly powerless of a part or of the whole of the body, from a dominant idea which has paralyzed or misdirected his volition. In such cases by altering the circulation, and breaking down the previous idea, and substituting a salutary idea of vigor and self-confidence in its place (which can be done by audible suggestions, addressed to the patient in a confident tone of voice, as to what *must* and *shall* be realized by the processes he has been subjected to), on being aroused, in a few minutes thereafter, with such dominant ideas in their minds, to the astonishment of themselves as well as of others, the patients are found to have acquired vigor and voluntary power over their hitherto paralyzed limbs, as if by a magical spell or witchcraft. Assuredly such cures are important as they are interesting and surprising, because such cases may resist ordinary modes of treatment for paralysis for an indefinite length of time; but still the *rationale* is simple enough, when viewed according to the principles which I have already explained, of the influence of an expectant dominant idea, *either exciting or depressing natural function, according to the faith and confidence of the patient.*"

On the 11th of August, the above patient called on me, accompanied by her mother. The patient was a tall, handsome, intelligent young lady, twenty-three years of age, five feet eight inches high, with figure well proportioned to her stature, so that when seated she had all the appearance of youth and vigor. When she attempted to walk, however, her paralytic condition of the left leg was very obvious, as she could only drag it along the floor at each step as far as the heel of the other foot, with the toes of the affected limb turned outwards. I had no difficulty in recognizing the nature of the case; so that I at once assured both mother and daughter that I would make very short work of that case. The mother said she would be glad if it turned out so; but she uttered this in a tone of voice which indicated that she was by no means equally sanguine on the point as I was. Having seated the patient in an easy chair, I hypnotised her, and extended her limbs, and acted on them so as to change the previously existing state of the muscles. In about ten minutes I aroused her, and requested her to walk across the room, which she was enabled to do, lifting the left foot from the floor, and carrying it forward before the other in the usual way; which she had not been able to do for months previously. The improvement, although she was still a little lame, seemed greatly to surprise both mother and daughter; more especially, from the apparent simplicity of the means by which it had been accomplished.

Next morning I repeated the operation, with still further improvement; and on the evening of the same day I operated again, after which she could walk up and down and around the room without the slightest appearance of lameness; and after a fourth operation, next morning, she could walk with the grace of a queen, or the agility of a sylph. Immediately after this operation, she rode to town, and there walked about through various shops and streets, as if she had never been lame at all. As the muscles of the other leg had also been somewhat affected, I recommended the patient to remain under treatment about a month, the more effectually to consolidate the cure; after which she returned home quite strong and active, and she remained so for twelve months.

During the summer of 1854, this patient had been so vigorous as to be able to climb the hills in the Highlands of Scotland as actively as her companions; but, during the autumn, from fatigue with a round of company, and anxiety about the health of a friend, her general health broke down; and, at the end of September, she had another paralytic seizure. As it had persisted for three weeks, she was brought to me once more; the mother of the patient expressing her fears, however, that I would not find hypnotism so successful as before, as her general health was so broken down on the present occasion. They arrived in Manchester at eight o'clock in the evening; and, as soon as they had had some refreshment, I told them that I intended to make the patient walk without lameness *before she went to bed.* The mother of the patient was quite incredulous; but I hypnotised, and acted as on the former occasion; and, in a quarter of an hour, the paralysis was quite gone, the patient walking without the slightest degree of lameness. After being hypnotised again next morning, she felt as vigorous as before the attack; and all the constitutional ailments her mother had been so anxious about speedily disappeared also under hypnotism, and the patient has kept quite well ever since.

Case 21.—On the 19th of June, 1854, I was consulted on the case of Miss R. Twelve months previously, she had had an attack of ophthalmia, which yielded to treatment so far that she was able to go out of doors in a month. She now had the misfortune to sustain a blow, from a pole falling upon the upper and left side of her head. Two or three days subsequent to this accident, she suffered severe pain from the blow, when suddenly she became quite blind of the eye on the same side, with dilation of the pupil. For this affection her medical attendant again subjected her to a course of treatment; and the result was, that in four months sight was partially restored to this eye. At the beginning of January, 1854, whilst reading the newspaper, this patient suddenly lost sight entirely of the other eye, with dilatation of its pupil, as had been the case previously with the other eye. Another surgeon was now consulted in the case; and a few days thereafter, whilst rising from the stooping posture near the fiire-place, the patient had the misfortune to strike the same part of the head against the mantel-shelf which had sustained the former injury from the falling of the pole, which blow against the mantel-shelf was immediately followed by a total loss of sight of the corresponding eye; and thus she required to be led about in a state of total blindness in both eyes. After treating the case for some time himself, the gentleman now in attendance, from a consideration of the obstinacy and importance of the case, recommended her to go to Dublin and place herself under the care of

Mr. Wilde, a celebrated oculist in that city. This was complied with, and she remained under the care of Mr. Wilde for six weeks, during which period she went through a course of very active treatment, with decided improvement; for the iris had become somewhat irritable on the application of light, and she was able to discern large objects, but could neither see to read nor write. She now returned home, where the same line of treatment was persevered with, under the supervision of the surgeon who sent her to Mr. Wilde. After she had been at home for some time, and finding the improvement had become stationary, this gentleman recommended her to try hypnotism, and furnished her with a letter of introduction to me, detailing the history of her case. On examination I found no apparent physical imperfection to account for the impaired vision; nor was there any pain about the head or eyes. The eyes had very much the appearance of an incipient case of amaurosis, only the pupils were not quite so much dilated. I suspected that the cause of the impaired vision was a want of sufficient irritability in the retina, and, if so, that hypnotism would very soon relieve her. My first object was to apply a test by which I might be enabled to ascertain what amount of benefit had resulted from my processes. On presenting the title-page of a book to her, with the largest and boldest letters in my room, I found she could not discern a single letter, notwithstanding there were some letters of a quarter of an inch long, and very bold open print. Having hypnotised the patient and directed the nervous power to the eyes, by wafting over them, and gently touching them occasionally, so as to keep up a sustained act of attention of the patient's mind to her eyes and the function of vision, she was aroused in about ten minutes. I now presented before her the title-page of the same book, when she instantly exclaimed, with delight and surprise, "I see the word commerce!" pointing to it. I told her she would see more than that presently; and in a little while she exclaimed, "I see commercial," then "I see dictionary;" and shortly after, "I see McCulloch," the name of the author; but she could see nothiug more. I told her that after a little rest, I felt assured she would see still smaller print, and, after a few minutes, she was able to read, "London, Longman, Green, and Longmans." Such was the result of my first process. After a second hypnotic operation, the next day, the patient could read, when first aroused, the whole of the title-page of a pamphlet; and, in about five minutes after, she sould read two lines of the text. After another operation, the same day, she could read the small close print in the appendix; and was able the same evening to write a letter home reporting progress, for the first time for twelve months. She only required two more hypnotic operations, when she was found able to read the smallest sized print in a newspaper; after which she left me, quite cured, and, as I have heard, she has continued well ever since.—*Ranking's Abstract.*

